# Simulation of Doxorubicin Delivery via Glucosamine(ethylene glycol) Carrier

**DOI:** 10.3390/ijms9112290

**Published:** 2008-11-21

**Authors:** Thongjun Pirawattana, Thongchai Srinophakun

**Affiliations:** 1 Chemical Engineering Department, Faculty of Chemical Engineering Kasetsart University, 50 Phaholyothin Road, Bangkok 10900, Thailand; 2 National Center of Excellence for Petroleum, Petrochemicals and Advanced Materials, Kasetsart University, Bangkok, 10900, Thailand

**Keywords:** Doxorubicin, Drug Delivery, Ethylene glycol, Glucosamine, Micelle

## Abstract

This article focuses on the molecular modeling of the release of doxorubicin from capsules composed of glucosamine(ethylene glycol) oligomers. Doxorubicin forms micelle structures with glucosamine(ethylene glycol), and the drug release mechanism can be studied through the modeling of oligomeric bond breaking under acidic, neutral, or basic conditions. Under these conditions, the activation energies were calculated to be 145.51, 135.78, and 287.60 kcal/mol, respectively, at the B3LYP/6-31G//PM3 level. Based on these values, doxorubicin can be released into acidic and neutral solutions but not into basic solution. Ethylene glycol chain length in glucosamine(ethylene glycol) also effects drug release. As the length of ethylene glycol increases, the amount of drug released increases under acidic conditions, but decreases under neutral and basic conditions. When the drug is released from glucosamine(ethylene glycol) oligomers, the drug molecule and glucosamine(ethylene glycol) molecules form a micelle structure. Studies found that, as the length of the ethylene glycol chains increases, the micelle structure is more easily formed. The ethylene glycol group can deliver doxorubicin to cancer cells in micelle form.

## 1. Introduction

Doxorubicin is an anthracycline ring antibiotic that is widely used as a cancer therapeutic. Common treatment side effects include nausea and vomiting, loss of appetite, diarrhea, and swelling [1], but development of a system capable of selectively delivering the drug to the intended target cells may be able to reduce these complications.

There are many approaches to drug delivery via drug/drug carrier combinations, such as encapsulation, hydrogel formation, nanoaggregation, and micellar delivery. For doxorubicin delivery, encapsulation and micellar delivery have received increased attention because this system can protect and carry the drug directed to its intended target. Brannon-Peppas and Blanchette [2] reported that tumor volumes in mice treated with an encapsulated drug conjugate were lower than tumor volumes in those treated with the conjugate alone. Janes *et al.* [3] modified chitosan nanoparticles to carry the doxorubicin. Wang *et al*. [4] encapsulated the chitosan, which has poor solubility in water, to increase the efficiency of drug delivery. Chan *et al.* [5] synthesized chitosan-g-poly(ethylene glycol) as an alternative drug delivery system with the intention that conjugation to poly(ethylene glycol) would increase the solubility of chitosan. There are several studies on micellar delivery of doxorubicin. Xiangyang *et al*. [6] used *N*-succinyl-*N*-octylchitosan micelles as doxorubicin carriers with effective anti-tumor activity. Zhang *et al*. [7] attached 10-hydroxycamptothecin, a hydrophobic anticancer drug, to amphiphilic *N*-alkyl-*N*-trimethylchitosan derivatives, resulting in increased drug solubility and controlled release.

Drug carriers usually have some chemical functional group used to detect their cancer cell targets. Yang *et al*. [8] used isocyanate terminated linear polyethylene glycol to carry drugs to cancer cells. Doxorubicin can be effectively delivered via conjugation to chitosan nanoparticles [9]. Son *et al*. [10] studied the tumor-targeting properties of glycol-chitosan nanoaggregates *in vivo* using fluorescein isothiocyanate-conjugated glycol-chitosan nanoaggregates and doxorubicin-conjugated glycol-chitosan (GC-DOX). Okabe *et al.* [11] studied *P*-glycoprotein efflux transporters, which confer drug resistance by decreasing the intracellular accumulation of anticancer drugs. Based on these previous experiments, we concluded that doxorubicin can be conjugated to a biopolymer that selectively targets cancer cells.

Quantum mechanical molecular simulation can be used to study drug delivery. Certain techniques, specifically semi-empirical and *ab initio* methods, are useful for simulating molecular interactions for drug delivery applications. Zavodinsky and Mikhailenko [12] simulated reactions between carbon nanoclusters and molecular oxygen using a semi-empirical PM3 method and found that reactivity depended on the structure of clusters and on the positions of the reactive carbon atoms. Propylene oxide structure optimization can be performed using semi-empiracal and *ab initio* methods [13]. The predicted bond lengths and bond angles agreed with experimental data. Simulations of the effect of hydrophobic molecules on aqueous solutions of amphiphilic block copolymers found that hydrophobic molecules will dissolve in micellar solutions [14]. This effect has also been investigated in detail by cryogenic transmission electron microscopy (TEM).

Cummins and Gready [15] used semi-empirical AM1 and PM3 methods to describe molecular interactions. Their method was applied to calculate the free energy of the enzymatic reduction of dihydrofolate (DHF) with nicotinamide adenine dinucleotide phosphate (NADPH) via *Escherichia coli* dihydrofolate reductase. The free energy change of this reaction agreed with the experimental results. *Ab initio* methods have used density functional theory (DFT), which was suitable for simulating reactions [16, 17].

In this work, the drug delivery doxorubicin with bis-polymer (chitosan) was studied for potential applications as an anticancer therapeutic. Both semi-empirical and *ab intio* methods were applied to optimize the structures of the relevant molecules and to find the possible mechanism of micelle formation.

## 2. Methodology

**Scheme 1.**
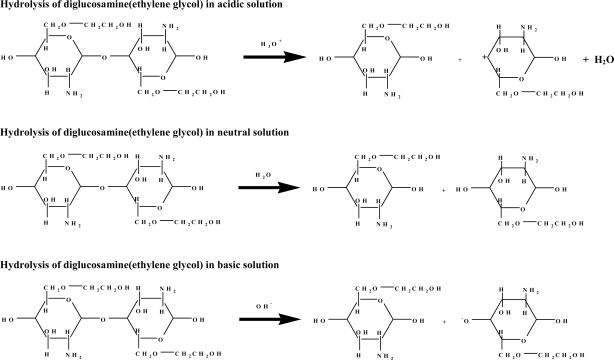
Hydrolysis of diglucosamine(ethylene glycol) under acidic, neutral, and basic conditions.

The encapsulation of doxorubicin within glucosamine(ethylene glycol) oligomers was simulated, and this process involved three steps. The first step was the structural optimization of both doxorubicin and glucosamine(ethylene glycol) using semi-empirical AM1 and PM3 methods and *ab initio* HF/6-31G methods. Optimization results show the potential of using glucosamine(ethylene glycol) as a drug delivery system. The oligomers form H-bonds with doxorubicin, and arrange the position of ethylene glycol at the outter site of micelle molecule in order to detect the target cells. The process of assembly was broken down into two parts, encapsulation and micelle formation. The second step was to simulate the release of doxorubicin from glucosamine(ethylene glycol) by considering the hydrolysis of diglucosamine(ethylene glycol) under acidic, neutral, and basic conditions (Scheme 1).

The third step was to study micelle formation with doxorubicin following the hydrolysis of diglucosamine(ethylene glycol) using B3LYP/6-31G//PM3 methods to simulate H-bond interactions. These simulations are calculated in gas phase and constant temperature at 298 K. All simulations were performed with Gaussian 03W and GaussView using computers equipped with Intel® Dual-Core Xeon™ 3.0 GHz processors and 2.0 GB of RAM. Input files will be available on http://www.ku.ac.th/~chemeng.

## 3. Results and Discussion

### 3.1. Structural optimization of doxorubicin and glucosamine(ethylene glycol)

Structures were optimized using molecular modeling methods. Frimand and Jalkanen [13] simulated propylene oxide with semi-empirical and *ab initio* methods, and their predictions were in good agreement with experimental data. Cummins and Gready [15] used semi-empirical quantum mechanical methods to describe molecular interactions relevant to the enzymatic cleavage of ribose ring phosphate groups from NADPH. The semi-empirical AM1 and PM3 predictions both agreed with experimental results.

In this study, the semi-empirical PM3 method was used to optimize the molecular geometries of doxorubicin and glucosamine(ethylene glycol). Bond lengths and charge structure were both considered geometric parameters and were optimized in this fashion.

### 3.1.1. Doxorubicin

**Figure 1.**
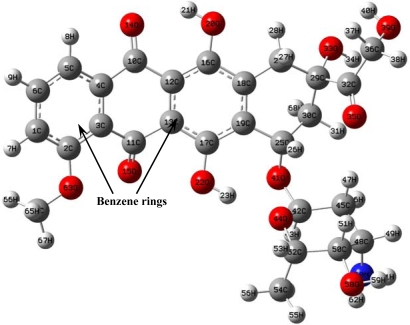
Optimized structure of doxorubicin.

The optimized doxorubicin structures obtained from semi-empirical AM1 and PM3 methods and from the *ab initio* HF/6-31G method were identical (Figure 1).

Some geometric parameters corresponding to this optimal structure are given in Table 1.The HF method predicted a more optimal structure than either the AM1 or the PM3 methods, but all three predictions demonstrated the same basic trends.

### 3.1.2. Glucosamine(ethylene glycol)

**Figure 2.**
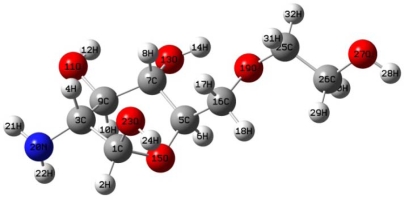
Optimized structure of glucosamine(ethylene glycol).

The optimal structure of glucosamine(ethylene glycol) was simulated using the same three aforementioned methods (AM1, PM3 and HF/6-31G). All three methods yielded nearly identical structures (Figure 2). The relevant geometric structural parameters from each method are given in Table 2.

All three methods predicted the same optimum structure, so the AM1, PM3, and HF methods can all be used for structure optimization. The optimized molecular structures for glucosamine(ethylene glycol) and doxorubicin were then used to study the two molecules’ interactions.

### 3.2. Drug release from capsules

The release of doxorubicin from its oligoglucosamine(ethylene glycol) capsule requires breaking the oligmeric bonds of oligoglucosamine(ethylene glycol). The probability of reaction can be estimated from the difference in total molecular energies between the products and the reactants. Simulations revealed that the capsule’s polymer bonds can be broken under acidic, normal, or basic conditions. These simulations involved two steps. First, molecular structures were optimized using the semi-empirical PM3 method. Second, the molecular energies were calculated via *ab initio* HF/B3LYP/6-31G method.

### 3.2.1. Acidic conditions

The following assumptions were made to simulate polymeric bond breaking under acidic conditions: (1) hydrolysis of diglucosamine(ethylene glycol) was used to represent cleavage of glucosamine(ethylene glycol) oligomers, and (2) H_3_O^+^ (hydronium ion) was used to represent acidic conditions.

**Figure 3.**
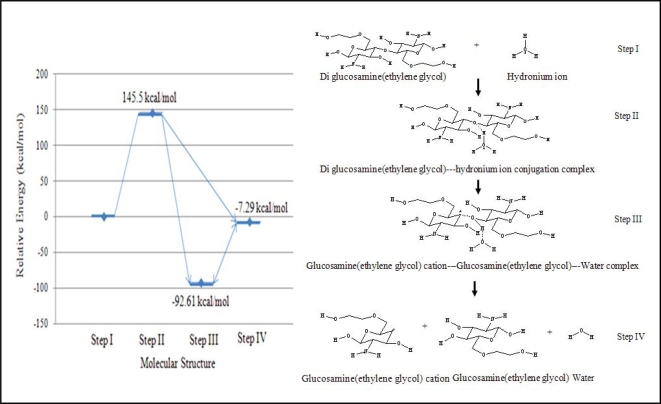
Relative energies of reaction intermediates during the acid hydrolysis of diglucosamine(ethylene glycol) calculated with B3LYP/6–31G//PM3. The reaction mechanism involves the four steps listed below. I. Diglucosamine(ethylene glycol) is attracted by hydronium ion. II. Diglucosamine(ethylene glycol) and the hydronium ion form a binary complex. III. The ternary product complex is generated containing glucosamine(ethylene glycol), a glucosamine (ethylene glycol) cation, and water. IV.The products dissociate into their final, stable states.

The reaction was also assumed to have four continuous steps. First, the hydronium ion complexes with diglucosamine(ethylene glycol) at the polymeric bond. Second, the hydrogen atom of the hydronium ion is attracted to the high electron density of the polymeric bond’s oxygen atom, forming the transition state complex. Third, the reaction generates a ternary complex among glucosamine(ethylene glycol), the glucosamine(ethylene glycol) cation, and water. Finally, these three product molecules separate. The relative energy of each step in the acid hydrolysis of diglucosamine(ethylene glycol) was calculated (Figure 3).

The acid hydrolysis reaction was simulated using B3LYP/6-31G//PM3 level calculations. The probability of reaction is related to the relative energy of the reaction. If the net change in energy for the reaction is negative, then product formation is favored. If the relative energy change is more negative, then the reaction products are more highly favored. The energies of each step were calculated relative to the molecular energy of step I, which represents the reactants. The energy of the transition state (step II) was 145.51 kcal/mol greater than that of the first step because the hydronium ion requires energy to react with glucosamine(ethylene glycol). Two distinct structures can be considered as the reaction’s final products. The first structure (step III) was the ternary product complex of glucosamine(ethylene glycol), a glucosamine(ethylene glycol) cation, and water, and this complex was predicted to have energy of –92.61 kcal/mol relative to the reactants in step I. The second structure (step IV), in which none of the product molecules interact, had a relative energy of –7.29 kcal/mol. Thus, the simulation predicted that the product complex from step III would be formed because it was of lower energy and was more stable than the non-interacting of product structure from step IV.

### 3.2.2. Neutral conditions

It was again assumed that diglucosamine(ethylene glycol) was an acceptable reactant surrogate for a glucosamine(ethylene glycol) oligomer. Since the simulated solution was of neutral pH, H_2_O was used as the nucleophile.

**Figure 4.**
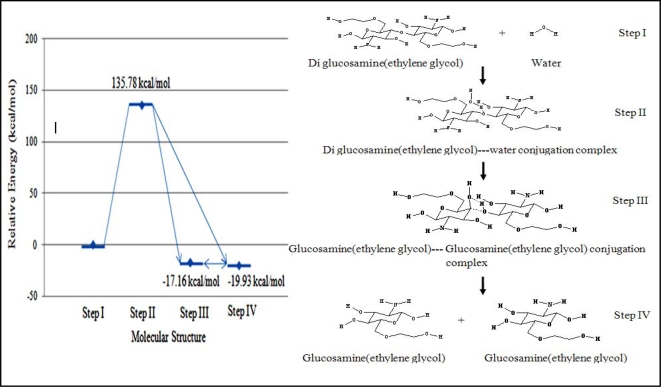
Relative energies of reaction intermediates during the hydrolysis of diglucosamine(ethylene glycol) under pH neutral conditions calculated with B3LYP/6–31G//PM3. The reaction mechanism involves four steps listed below. I. Diglucosamine(ethylene glycol) attracts the water molecule. II. Diglucosamine(ethylene glycol) and water form a binary complex. III. The binary product complex is generated containing two molecules of glucosamine(ethylene glycol). IV. The product complex dissociates into two molecules of glucosamine(ethylene glycol).

The molecular energy of each step in the reaction mechanism was calculated relative to that of step I. The relative energy of the transition state was 135.78 kcal/mol. Both steps III and IV were considered for the final product. The first structure (stage III), which had a relative energy of –17.16 kcal/mol, was the complex product containing two molecules of glucosamine(ethylene glycol). The second structure (stage IV), which had a relative energy of –19.93 kcal/mol, was the dissociated glucosamine(ethylene glycol) molecules (Figure 4).

### 3.2.3. Basic conditions

Again, diglucosamine(ethylene glycol) was assumed to be an acceptable reactant surrogate for a glucosamine(ethylene glycol) oligomer. As hydrolysis was simulated under basic conditions, OH^−^ was used as the nucleophile.

Two different hydrolysis products can form under basic conditions. Either the hydroxide ion reacts with the polymeric bond forming the desired products or it deprotonates the molecule. In the case of the former reaction, the high electron density on the oxygen of the polymer bond repels the hydroxide ion, hindering nucleophilic attack. In the case of the latter reaction, the hydroxide ion removes a proton from the ethylene glycol moiety of diglucosamine(ethylene glycol) forming a dimeric anion.

### 3.2.3.1. Hydroxide ion hydrolysis of the polymeric bond

**Figure 5.**
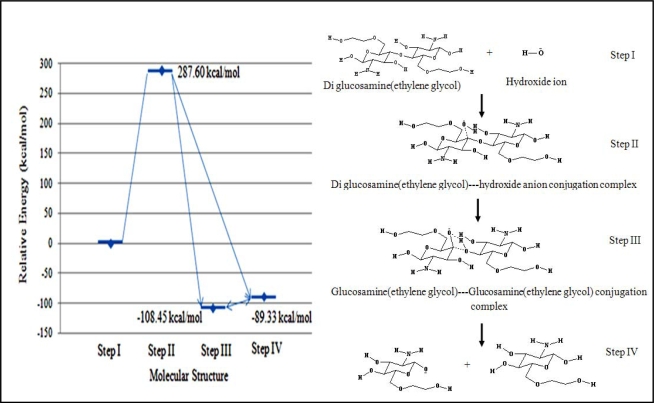
Relative energies of reaction intermediates during the basic hydrolysis of diglucosamine(ethylene glycol) calculated with B3LYP/6–31G//PM3. The reaction mechanism involves the four steps listed below. I. Diglucosamine(ethylene glycol) attracts the hydroxide ion. II. Diglucosamine(ethylene glycol) forms a complex with the hydroxide ion. III. The binary product complex is generated containing glucosamine(ethylene glycol) anion and a glucosamine(ethylene glycol). IV. The binary complex dissociates into one molecule of glucosamine(ethylene glycol) anion and a glucosamine(ethylene glycol).

Under basic conditions, a hydroxide ion can act as a nucleophile to hydrolyze the diglucosamine(ethylene glycol) polymeric bond. The relative energy of the transition state was 287.60 kcal/mol. Both steps III and IV were considered for the final product. The first structure, which had a relative energy of –108.43 kcal/mol, was the binary product complex containing two molecules of glucosamine(ethylene glycol). The second structure, which had a relative energy of –89.33 kcal/mol, was dissociated complex consisting of a molecule of glucosamine(ethylene glycol) and glucosamine(ethylene glycol) anion (Figure 5).

The high activation energy of this reaction suggested that this reaction cannot proceed at an appreciable rate. The high relative energy of the transition state was attributed to the repulsive effect of the high electron density on the polymeric linkage.

### 3.2.3.2. Hydroxide ion deprotonates ethylene glycol group

**Figure 6.**
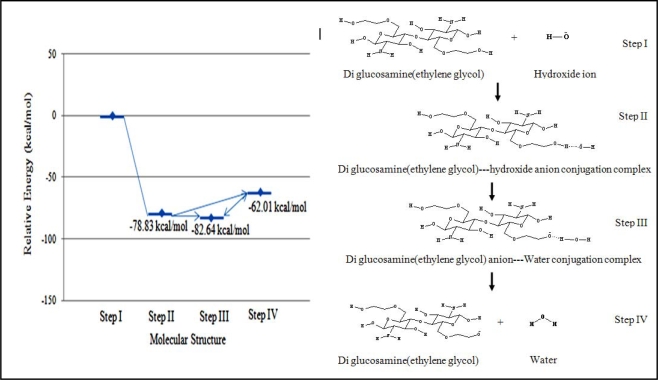
Relative energies of reaction intermediates during the deprotonation of diglucosamine(ethylene glycol) calculated with B3LYP/6–31G//PM3. The reaction mechanism involves the four steps listed below. I. Diglucosamine(ethylene glycol) attracts the hydroxide ion. II. Diglucosamine(ethylene glycol) forms a complex with the hydroxide ion. III. A binary product complex is generated containing a diglucosamine(ethylene glycol) anion and a molecule of water. IV. The product complex dissociates into a diglucosamine(ethylene glycol) anion a molecule of water.

The hydroxide can also deprotonate the ethylene glycol moiety of diglucosamine(ethylene glycol) under base conditions and the relative energy of each mechanistic step was calculated (Figure 6).

The relative energy of the transition state was –78.83 kcal/mol. This implied that this reaction would dominate under basic conditions because it was much faster than hydrolysis. Two possible products were considered to follow the transition state. The first structure, which had an energy of –82.64 kcal/mol, was the product complex containing a diglucosamine(ethylene glycol) anion and a molecule of water. The second structure, which had an energy of –62.01 kcal/mol, was the dissociated diglucosamine(ethylene glycol) molecule.

The results for all three conditions (acid, neutral, and basic) are summarized in Table 3. The activation energy was highest for the basic hydrolysis reaction (Basic I). With the exception of neutral hydrolysis conditions, the product complex states (all mechanisms, step III) had relative energies lower than those of the dissociated products (all mechanisms, step IV). This implied that the molecules most likely stay closely associated following reaction. From these stages (step III), the complex molecules can move apart from each other and stay in stable form with optimum structure (stage IV). In the case of a competing side reaction under basic conditions (basic II), the high electron density at the ethylene glycol oxygen is insufficient to repel the hydroxide anion and prevent deprotonation but at hydrogen is mild electron density, which occurs very rapidly. The hydroxide anion is likely to form at hydrogen position of ethylene glycol than of polymer bond.

These predictions agreed with the experimental results of Oungbho *et al*. [18], who found that drug release from chitosan was much more favorable under acidic conditions than under neutral conditions. Under basic conditions, the hydroxide ion is difficult to attack at polymer bond because of very high electron density but deprotonation of the ethylene glycol is much faster than hydrolysis, and so drugs cannot be released from a glucosamine(ethylene glycol) capsule in this manner.

### 3.3. Micelle formation

Glucosamine(ethylene glycol) and doxorubicin can form complex micellar structures via hydrogen bonding. A molecule of glucosamine(ethylene glycol) consists of both hydrophobic (glucosamine) and hydrophilic (ethylene glycol) parts. The glucosamine moiety engages in hydrogen bonds with doxorubicin, while the ethylene glycol moiety allows the complex to dissolve in water.

Micelle formation was simulated using B3LYP/6-31G//PM3 methods in which the partial charge distributions of doxorubicin and glucosamine(ethylene glycol) were considered. PM3 was used to optimize the structures of doxorubicin and glucosamine(ethylene glycol). As one can notice from figures, the red numbers and circles are H-bond between doxorubicin and glucosamine (ethylene glycol).

**Figure 7.**
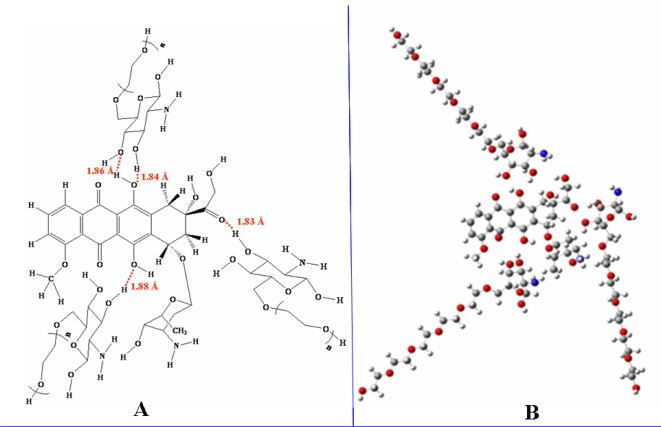
Simulated doxorubicin micelle using B3LYP/6–31G//PM3 methods. (A) Micellar structure. where n = 1; glucosamine-mono(ethylene glycol) n = 2; glucosamine-di(ethylene glycol) n = 3; glucosamine-tri(ethylene glycol) n = 4; glucosamine-tetra(ethylene glycol) n = 5; glucosamine-penta(ethylene glycol) (B) Molecular structure of glucosamine-penta(ethylene glycol)

The charge structure of doxorubicin is obviously negative on O and N. Hydrogen bonds formed between the O-H and N-H groups of doxorubicin and glucosamine(ethylene glycol). In this simulation, the distribution of partial charges was calculated from electron density. The negative partial charges at the oxygen atoms of doxorubicin interacted with the positive partial charges of the protons of glucosamine(ethylene glycol). The charge consideration is calculated from high negative charge on O of doxorubicin and high positive charge on H of glucosamine(ethylene glycol) interaction. Predictions indicated that the doxorubicin structure contained three points of high electron density, and the glucosamine(ethylene glycol) possessed hydrogen atoms with low electron density. Micelle formation was hypothesized to require precisely controlled amounts of both the glucosamine and the ethylene glycol moieties. The stability of micelle is analyzed from the length of ethylene glycol chain and amount of glucosamine(ethylene glycol) to form micelle structure. The amount of ethylene glycol present was changed by the varying the length of the *n*-ethylene group in glucosamine(*n*-ethylene glycol) between n=1 and n=5. The simulated micelle is depicted in Figure 7.

**Figure 8.**
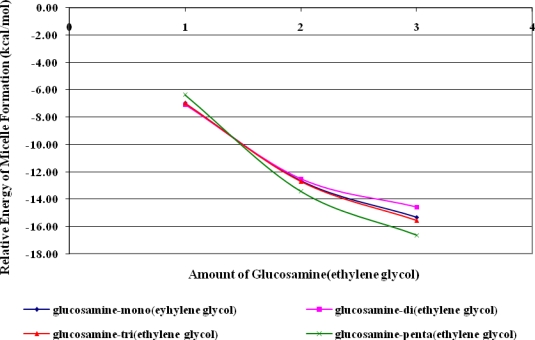
Relationship between the relative energy of micelle formation and the amount of glucosamine(ethylene glycol) present.

The energy of micelle formation was also related to the amount of glucosamine(ethylene glycol) present (Figure 8). Increasing the relative amount of ethylene glycol in glucosamine(*n*-ethylene glycol) presumably prevented water from becoming trapped within the hydrophobic part of the micelle and interfering with hydrogen bonding. This exclusion of water decreased the relative energy of micelle formation thereby facilitating the process. These simulations agreed with the results of Xiangjang [6] and Mitra [9]. The relative energy of micelle formation also decreased when the number of glucosamine(ethylene glycol) molecules was increased because additional hydrogen bonding between doxorubicin and glucosamine-(ethylene glycol) provided additional stabilization. The amount of glucosamine(ethylene glycol) played a larger role in micelle stabilization than did the length of the *n*-ethylene glycol side chain because more hydrogen bond makes micelle structure more stable. Once formed into a micelle, the ethylene glycol moiety shows the potential to be a doxorubicin delivery to cancer cells [5, 20–22].

## 4. Conclusions

The hydrolytic release of doxorubicin from a glucosamine(ethylene glycol) oligomer capsule was simulated under acidic, neutral, and basic conditions. Using diglycosamine(ethylene glycol) as a surrogate substrate, hydrolysis was predicted under acidic and neutral conditions but not under basic conditions. Thus, an acidic environment, such as the stomach, may break the encapsulating polymer bonds. Deprotonation is favored over hydrolysis under basic conditions, so the capsule will not be broken in this case.

Micelles formation from doxorubicin and glucosamine(*n*-ethylene glycol) depended on the length of the ethylene glycol side chain. According to B3LYP/6-31G//PM3 predictions, lengthening the ethylene glycol chain stabilized the resulting micelles, thereby facilitating their formation. The number of glucosamine(ethylene glycol) molecules included in the simulation was varied from one to three, and increasing the number of molecules also facilitated micelle formation. Thus, glucosamine(ethylene glycol) and doxorubicin will form complex micellar structures capable of acting as drug delivery systems.

The capsule polymer used to deliver doxorubicin should possess a sufficiently long ethylene glycol chain to promote micelle formation. The length of the ethylene glycol chain also had an effect on polymer hydrolysis. In an acidic solution, increasing of the length of the ethylene glycol side chain facilitated hydrolysis because the side chain pulled H_3_O^+^ from solution, thereby reducing the relative energy of the transition state. If, however, the ethylene glycol chain was too long, it reacted with the polymeric bond, forming (diglucosamine-tri(ethylene glycol)). In neutral and basic solutions, as the length of ethylene glycol chain increased, the polymeric bonds became more resistant to hydrolysis because the ethylene glycol side chain sterically hindered nucleophilic attack.

## Figures and Tables

**Table 1.** Geometric parameters of optimized doxorubicin structure.

	Semi-empirical		*Ab initio*
Bond length	AM1	PM3	HF/6–31G
C64 - H66	1.117	1.096	1.082
C64 - O63	1.424	1.406	1.429
C2 - O63	1.376	1.375	1.359
C16 - O20	1.374	1.363	1.363
C3 - C11	1.48	1.495	1.492
C19 - C25	1.504	1.511	1.518
C29 - C32	1.533	1.558	1.532
C48 - N60	1.45	1.488	1.451
O20 - H21	0.971	0.962	0.959
N60 - H61	0.999	0.998	0.996
C11 = O15	1.233	1.213	1.215
C1 = C2	1.405	1.404	1.389
**Dihedral angle**			
H65-C64-O63-C2	–65.1	–66.2	–66.2
C2-C3-C11-O15	28.6	41.8	31.7
C13-C17-O22-H23	–167	178.6	145.4
C50-C48-N50-H61	–143.1	–162.3	–175.9
O35-C32-C36-O39	131.4	106.6	126.5
H9-C6-C5-H8	–0.4	0.3	0.1
C5-C4-C10-O14	–20	–21.7	–9.8
O28-C16-C18-C24	0.5	0.1	0.7
C10-C4-C3-C11	–3.5	–6.6	–7.8

**Table 2.** Geometrical parameters of optimized glucosamine(ethylene glycol) structure.

	Semi-empirical		*Ab initio*
Bond length	AM1	PM3	HF/6–31G
C3 - H4	1.134	1.121	1.079
C1 - C3	1.552	1.555	1.534
C3 - C9	1.548	1.549	1.529
C1 - O23	1.409	1.401	1.42
C1 - O15	1.415	1.41	1.426
C3 - N20	1.445	1.482	1.452
N20 - H22	1.002	0.999	0.998
O11 - H12	0.967	0.948	0.953
**Dihedral angle**			
H21-N20-C3-1C	169.9	164.2	157.9
O11-C9-C7-O13	–56.9	–63.7	–55.3
C7-C5-C16-O19	–62.5	–73.1	–56.8
O19-C25-C26-O27	175.8	175.4	–179.4
C1-O15-C5-C16	93.9	89	104.4
H22-H20-C3-4H	173.4	166.7	174.7
H2-C1-15O-5C	175.6	166.7	174.2
H6-5C-16C-19O	58.6	49.8	63.5
H8-7C-5C-16C	–30.1	–20.1	–39.8

**Table 3.** Comparison of possible reaction pathways.

Simulated Mechanism	Step I	Step II	Step III	Step IV
	kcal/mol			
Acidic	0	145.50	–92.61	–7.29
Neutral	0	135.78	–17.16	–19.93
Basic I	0	287.6	–108.45	–89.33
Basic II	0	–78.83	–82.64	–62.01
